# The impact of religiosity on dietary habits and physical activity in minority women participating in the Health is Power (HIP) study

**DOI:** 10.1016/j.pmedr.2016.12.012

**Published:** 2016-12-21

**Authors:** Serene Ansari, Erica G. Soltero, Elizabeth Lorenzo, Rebecca E. Lee

**Affiliations:** a School of Allied Health Sciences, Baylor College of Medicine, One Baylor Plaza, MS: BCM115, Houston, TX 77030, United States; b Center for Health Promotion and Disease Prevention, College of Nursing and Health Innovation, Arizona State University, 300 North 3rd Street, Phoenix, AZ 85004, United States

**Keywords:** Exercise, Food, Nutrients, Food habits, Religion, Ethnic groups, Female, African-Americans, Hispanic Americans, Health status disparities, Community health planning

## Abstract

African American (AA) and Hispanic/Latina (HL) women report lower rates of physical activity (PA) and poorer dietary habits compared to their white counterparts. Religiosity can act as a protective factor for health; however, the relationship between religiosity, PA, and diet is unclear. This study aimed to investigate the influence of religiosity on PA and fruit and vegetable (FV) and fat consumption in minority women. Health is Power (HIP) was a 6-month intervention where participants (AA: 63%; HL: 37%) were randomized to a PA or FV group. Questionnaires assessed religiosity at baseline and PA, FV and fat consumption at baseline and post-intervention. Hierarchical linear regression models were used to investigate religiosity as a predictor of change in PA, FV and fat, while controlling for demographics. AA women had significantly higher religiosity scores (M = 44.15, SD = 10.66) compared to H/L women (M = 35.11, SD = 12.82; *t*(251) = 5.86, *p* < 0.001). Across both groups, PA increased by 15%, FV intake increased by 27%, and consumption of calories by fat decreased by 5%. Religiosity was not a significant predictor of PA or diet (*p* < 0.05). The results of this study found no association between religiosity and change in PA and diet. More longitudinal studies are needed to explore the role of religiosity in the health of minority women.

## Highlights

• Religiosity or spirituality may influence some health behaviors in minority women.
• Religiosity or spirituality did not influence adoption of physical activity.
• Religiosity or spirituality did not influence adoption of healthy eating.

## 1 Introduction

It is well-established that the combination of lack of physical activity and poor dietary habits increases the risk of obesity and related diseases (Lee et al., 2011a). Physical inactivity is particularly prevalent among women and minorities (Lee & Cubbin, 2009). African American (AA) and Hispanic/Latino (HL) women are less likely to meet physical activity (PA) or fruit and vegetable (FV) recommendations compared with whites (Lee et al., 2011a, Lee and Cubbin, 2009, Facts about Physical Activity, 2015).

Religiosity and spirituality (R/S) has been shown to positively affect health (Koenig, 2012, Tan et al., 2013). R/S may impact health through individual implementation of health-promoting religious acts and beliefs, religious prohibitions against unhealthy behaviors, and other religious laws (Tan et al., 2013, Hart et al., 2006). R/S may also impact health as a R/S community can provide supportive social connections that can reinforce behaviors, increase community trust and involvement, and potentially allow for the flow of health information (Koenig, 2012, Gillum, 2006, Underwood, 2006). R/S has been positively associated with well-being, self-esteem, reduced risk of all-cause mortality, and lower rates of lifestyle-related diseases such as hypertension, cardiovascular disease, and cancer (Koenig, 2012, Tan et al., 2013). Of the few studies that have examined this relationship, some found that R/S was associated with more PA and a healthier diet, while other studies found no relationship (Gillum, 2006). Longitudinal studies are needed to explore the relationship between R/S and improvement in PA and dietary habits, particularly in minority women (Lee et al., 2011a, Lee and Cubbin, 2009, Mama et al., 2014, Lee et al., 2011b, Lee et al., 2012).

The majority of previous studies have been cross-sectional, do not investigate whether those with more R/S who participate in behavioral interventions have greater success at behavior change, and typically do not include ethnic minority women. Many studies have relied on measures that have not demonstrated adequate reliability and validity. In addition, some studies do not control for demographic variables, which can also affect the outcome of the relationship being tested (Koenig, 2012, Tan et al., 2013, Hart et al., 2006). Therefore, further research in a longitudinally designed model with standardized measurement tools focused on ethnic minority women is necessary to establish guidelines for behavioral interventions in the future. The purpose of this study was to investigate the influence of spirituality and religious involvement on the adoption of PA and healthful dietary habits of ethnic minority women participating in a behaviorally based intervention using a longitudinal design, hypothesizing that there would be a positive association between degree of R/S and the increase in PA and consumption of FV, and decrease in consumption of fat. Improved understanding of these relationships could improve interventions to address the distressing disparities in the health of ethnic minorities.

## 2 Methods

### 2.1 Design and setting

Health is Power (HIP) was a multisite, randomized, controlled trial to increase PA and improve dietary habits in AA and HL women. HIP was implemented in Houston and Austin, Texas from 2006 to 2008. Women were recruited using a variety of strategies including face-to face announcements at community meetings and events, advertisements in local media (Lee et al., 2016). Participants were randomly assigned to a PA group or vegetable and fruit comparison group. Both intervention groups met six times over a six-month period and were behaviorally based, using group cohesion techniques to foster cohesion, cooperation and friendly competition specific to either PA or vegetable and fruit consumption (Lee et al., 2011a, Lee et al., 2012). All HIP study assessments, measures, and procedures were approved by the Committee for the Protection of Human Subjects at the University of Houston, and participants provided written informed consent to participate (HRP-503a) (Lee et al., 2011b, Lee et al., 2012).

### 2.2 Participants

Recruited women (*N* = 410; Houston = 311, Austin = 99) self-identified as AA (63%) or HL (37%) were between the ages of 25 and 60 years old, not pregnant or planning to become pregnant within the next 12 months, and apparently healthy. After women were recruited, they completed a run in procedure as described below to avoid assigning women to groups who might be more likely to drop out, resulting in a sample of 310 women assigned to groups. A total of 198 women completed post-intervention measures, representing 64% of those assigned, which compares favorably to other studies of ethnic minority women (Lee et al., 2016). Of these, 132 had complete data on all measures, and these cases were used for analyses.

### 2.3 Individual measures

Women completed measures of PA, FV, and fat intake at the T1 assessment. These women were given a take home packet containing health questionnaires assessing R/S as part of the run-in procedure. The International Physical Activity Questionnaire (IPAQ) long form was used to measure self-reported walking, leisure-time, and total physical activity over the last 7 days. PA was reported as days per week and minutes and/or hours per day and was converted to metabolic equivalent minutes (MET-minutes) (Lee et al., 2011b, Lee et al., 2012). Dietary habits were measured using the National Cancer Institute Fruit and Vegetable Screener and Fat Screener. Fruit and vegetable consumption was reported as frequency and amount consumed each time over the last month. The Fat Screener measures an individual's usual dietary intake of percent calories from fat (Lee et al., 2011b, Lee et al., 2012). R/S was measured using the National Institute of Aging/Fetzer Short Form for the Measurement of Religiousness and Spirituality. This multidimensional tool is brief, measures traditional religiousness and non-institutional spirituality, is appropriate for Judeo Christian populations, and was developed with the specific goal of assessing the association between R/S and health. After 6 months of intervention activities, women returned to complete a post-intervention (T2) assessment 6 months later (Lee et al., 2011b, Lee et al., 2012). The R/S questionnaire was administered at T1 baseline only.

### 2.4 Statistical analysis

Descriptive analyses were performed to describe individual health characteristics. Bivariate correlations were assessed to determine whether there were statistically significant correlations among R/S, PA, FV and fat consumption. Items on the R/S instrument were reverse coded and transformed to a z score before being summed into a continuous variable. Surveys with more than 3 items missing were not included. The minimum to maximum possible score ranged between 19 and 103 (Idler et al., 2003).

In order to determine uniqueness of R/S as a significant predictor for improvement in PA and dietary habits, while controlling for covariates, five linear regression analyses were conducted, one for each of the three PA variables and one for each of the two dietary habits variables with the post-intervention (T2) behavioral measure as the dependent variable. For each model, in the first block, the baseline (T1) behavioral measure was entered. Next in the second block, the potential demographic variables (educational attainment, household income, intervention site, age, and ethnicity) were entered. In the third block, the R/S variable was added. All analyses were performed using IBM statistical software SPSS version 22.0, and a *p* value of < 0.05 was used as the criterion for all statistical testing.

## 3 Results

Of the participants, 132 provided complete data for this study as described above. Most participants (63%) were African American (AA), and 37% identified as Hispanic/Latina (H/L). Women had a mean age of 45; 32% identified as Protestant, 28.6% as Catholic, 9.4% as Jewish, 27% as Another, and 3% as None. Eighty-nine percent had some college education and 49% reported an income 401% or greater above the Federal Poverty Level for a family of four. Women scored an average of 41.6 (SD = 11.3) on R/S. Demographic characteristics of the study sample are presented below in Table 1.

At post-intervention, physical activity levels increased by 15%, FV intake increased by 27% and fat consumption decreased by 5%. Physical activity and dietary habit outcomes pre- and post-intervention are presented in Table 2, below. Bivariate correlations revealed that there were no significant relationships between variables of interest. Linear regression models were unable to identify R/S as a statistically significant and unique predictor of improvement in PA, FV or fat consumption, after adjusting for demographic variables.

## 4 Discussion

The HIP study is among the first to longitudinally investigate how R/S may influence adoption of PA and dietary habits among AA and H/L women participating in behaviorally based health interventions (Lee et al., 2012, Idler et al., 2003). Across both intervention groups, women reported increased PA and FV consumption, as well as decreases in calories consumed from fat. This showed that the HIP intervention itself had a positive impact on the participants' health behaviors as has been previously reported (Lee et al., 2012). However, secondary analyses investigating the hypothesized relationship that R/S might enhance behavioral adoption found no significant relationship, suggesting that R/S may not promote adoption of PA or improvement in dietary habits.

These longitudinal findings are consistent with previous cross-sectional studies that showed R/S has been inconsistently associated with PA and dietary habits. (Koenig, 2012, Idler et al., 2003, Watkins et al., 2013, Kim and Sobal, 2004, Cline and Ferraro, 2006) Koenig et al. further reviewed 36 studies examining associations between weight and R/S involvement and found 39% with a positive relationship, and 19% as an inverse relationship (Koenig, 2012). In another publication, with results from the Health and Retirement Study, 8422 cancer survivors including both African Americans and Hispanic, found that people who were more religiously oriented also showed greater likelihood of obesity and lesser likelihood of self-reported regular weekly exercise (Nathenson & Wen, 2012). Underwood et al. published a study that demonstrated those participants who identified as “very or moderately religious” were least likely to report diets with consumption in accordance to nutrition guidelines, unlike those who identified as “self-directive or slightly spiritual.” (Underwood, 2006).

Others have postulated that religious individuals are, in fact, at greater risk for medical illness due to their higher BMIs when compared to the non-religious population (Cline & Ferraro, 2006). It may be that some religions may be more or less protective. For example, most fundamentalist religions emphasize the abstinence of tobacco and alcohol, but do not focus on the need for moderation in food consumption. Food might be considered an admissible source of pleasure. Thus, gluttony becomes an accepted vice. Religious and cultural practices common among some ethnic minority groups may include eating, which could contribute to poor health depending on food selections and preparation styles. Tan et al. posited that while religion may not necessarily cause obesity, it may be considered a safe haven to those who would be otherwise stigmatized for being overweight or unhealthy (Tan et al., 2013). Newer research adds that in the growing age of technology, those who participate in regular “religious media practice” are largely sedentary when acquiring their daily dose of religion from the TV and internet, in isolation at home and with access to food (Nathenson & Wen, 2012). Lower weight among more those with more R/S appears only in the Amish, Jewish, and Buddhist, and those who are white, older, and more educated (Koenig, 2012). The current findings and those from other studies suggest that greater specificity in the measurement of religion and religious practices may be needed to understand when R/S can be protective or destructive toward maintaining good health habits. As well, faith based and faith placed interventions may be more successful at incorporating elements from specific faiths that can help members change their health habits in the context of that faith (Fitzgibbon et al., 2005, Gutierrez et al., 2014, Wilcox et al., 2013).

Strengths of the study include use of a sample representing ethnic minority women who have been understudied in this line of research. Additional strengths include the longitudinal design, diverse sample, and focused intervention with ethnic minority women. Although data were self-reported (Lee et al., 2011b), outcome variables relied on reliable and well-validated measures. It is possible that when answering questions about R/S participants may have lacked introspective ability or may have found it difficult to rate abstract concepts on a scale (Tan et al., 2013). Future studies that use objective measures such as official logs for church attendance, continuously monitored physical activity (e.g., pedometers, accelerometers) or food diaries may produce different findings. One of the major barriers to research on R/S has been the lack of generally accepted measurement protocols for this construct. Unlike other surveys, this R/S tool does not use a single-item scale like religious attendance only, to determine level of R/S (Gillum, 2006, Idler et al., 2003). Although the measure used in this study was comprehensive and reliable, there was a relatively small number of items for each domain, which in turn may have caused scores to be less stable and prone to measurement error (Idler et al., 2003). Future studies could also develop a R/S tool that is more precise in its measurement such that it focuses on PA and dietary habits, specifically. On average, the women in this study reported higher socioeconomic status compared to the general population. Missing data may have presented the possibility of selection bias (Tan et al., 2013). This sample had a higher income and was more highly educated than the population in their counties of residence (Lee et al., 2011b).

Although the R/S tool distinguished between denominations, no further analysis was performed with respect to these specific subsets. This sample is only representative of the southern U.S. Future studies should measure participants across the nation. Sample size may also benefit from being increased, as larger sample sizes naturally produce more generalizable results (Gillum, 2006).

In light of the findings of this study revealing the potentially negative impact of R/S on adoption of healthy habits, and given the centrality of the church in many ethnic minority communities, there is a unique opportunity for the development of targeted, faith-based interventions to tackle the aforementioned health disparities. Policy implications of this trend may include utilizing the church to advocate a healthy lifestyle, sanction against negative behaviors, provide social support, and have a positive impact on self-esteem (Nathenson & Wen, 2012). As it has been successful in the past, dietary interventions designed to increase fruit and vegetable intake among church members should be incorporated. Interventions should include education regarding portion control and preparation methods. For those of a lower socioeconomic position, this may include actual transportation to stores, and increasing health literacy to avoid adverse health outcomes.

## 5 Conclusions

R/S can be a vital feature of many people's lives, and literature has shown that they can affect health and health outcomes (Tan et al., 2013); however, this longitudinal study did not show a relationship between R/S and health outcomes. Additional research using longitudinal designs, embedded faith messages, and reliable measurement tools may more definitively delineate the role of R/S on adoption of health behaviors. Employing the knowledge we gain from this and future scientific studies to understand the particular contexts of R/S that are influential in shaping health behaviors will aid in narrowing the gaps of health disparities.

## Funding source

This study was supported by a grant from the National Institutes of Health's National Cancer Institute (1R01CA109403) awarded to Dr. Lee.

## Conflict of interest statement

The authors declare that there are no conflicts of interest.

## Transparency document

Transparency document. Image 1

## Figures and Tables

**Table 1:** Participant demographic characteristics in the Health is Power study.

Demographic variable *N* = 403	Mean (SD)
Age	45.2 (9.35)
Houston	74.6 (304)
Austin	25.4 (99)
Ethnicity (%)	
African American	62.7 (252)
Hispanic/Latina	37.3 (149)
Religion (%)	
Protestant	32.1 (129)
Catholic	28.6 (115)
Jewish	9.4 (38)
Another	27.4 (110)
None	2.6 (10)
Education (%)	
Some college or college graduate	88.7 (346)
Greater than 400% over the poverty line	49.3 (182)

**Table 2:** Pre- and post-intervention means and standard deviations for primary outcomes across the sample.

	Pre-intervention M (SD)	Post-intervention M (SD)
Physical activity (MET-min/week)		
Total PA	2794 (3754)	3207 (2912)
Leisure time PA	325 (661)	757 (1011)
Walking	846 (1772)	910 (1053)
Dietary habits		
Fruit & vegetable intake (servings/day)	2.92 (2.69)	3.72 (2.80)
Fat intake (%kcal/day)	31.85 (3.87)	30.34 (3.63)
